# Comparative activity of carbapenem testing (the COMPACT study) in Turkey

**DOI:** 10.1186/1471-2334-12-42

**Published:** 2012-02-16

**Authors:** Hakan Leblebicioglu, Nedim Cakir, Mustafa Celen, Halil Kurt, Hakan Baris, Joerg Laeuffer

**Affiliations:** 1 Ondokuzmayis University, Kurupelit Kampüsü, PK 55139, Kurupelit, Samsun, Turkey; 2 Dokuz Eylül University Infectious Disease Clinic, 35340, İnciralti İZMİR, Izmir, Turkey; 3 Dicle University Infectious Disease Clinic, 21280, Diyarbakir, Turkey; 4 Ankara University Infectious Disease Clinic, PK 06230, Altindağ, Gündoğdu Ankara, Turkey; 5 Janssen-Turkey, Ertürk Sok, Keçeli Plaza No: 13 34810, Kavacik, Beykoz, Istanbul, Turkey; 6 Janssen-EMEA, Sihlbruggstrasse 111, 6340, Baar, Switzerland

## Abstract

**Background:**

Recent evidence indicates that Gram-negative bacterial pathogens, the most common of which are *Pseudomonas *spp., *Enterobacteriaceae*, and *Acinetobacter baumannii*, are frequent causes of hospital-acquired infections. This study aims to evaluate the in vitro activity of doripenem and comparator carbapenem antibiotics against Gram-negative clinical isolates collected from COMParative Activity of Carbapenem Testing (COMPACT) study centres in Turkey.

**Methods:**

Ten centres in Turkey were invited to submit *Pseudomonas aeruginosa*, *Enterobacteriaceae*, and other Gram-negative isolates from intensive care unit (ICU)/non-ICU patients with complicated intra-abdominal infections, bloodstream infections, or nosocomial pneumonia, including ventilator-associated pneumonia, between May and October 2008. Susceptibility was determined by each centre using E-test. A central laboratory performed species confirmation as well as limited susceptibility and quality-control testing.

**Results:**

Five hundred and ninety six isolates were collected. MIC_90 _values for doripenem, meropenem, and imipenem, respectively, were 32, ≥ 64, and ≥ 64 mg/L against *Pseudomonas *spp.; 0.12, 0.12, and 0.5 mg/L against *Enterobacteriaceae*; and ≥ 64 mg/L for each against other Gram-negative isolates. In determining the susceptibility of hospital isolates of selected Gram-negative pathogens to doripenem, imipenem, and meropenem, we found that against all pathogens combined, the MIC_90 _for ICU compared with non-ICU isolates was higher.

**Conclusions:**

Doripenem showed similar or slightly better activity than meropenem and better activity than imipenem against the Gram-negative pathogens collected in Turkey.

## Background

Modified treatment practices towards improving in-hospital patient care and reducing the development and spread of resistant strains begin with the surveillance of known infection-causing pathogens. Isolates of particular importance within the hospital setting are *Pseudomonas *spp. and *Enterobacteriaceae*, as well as other Gram negatives such as *Acinetobacter baumannii *(*A.baumannii*). Recent evidence indicates these pathogens are common causes of infection, including complicated intra-abdominal infection (cIAI), blood stream infection (BSI), and nosocomial pneumonia (NP). *Escherichia coli *(*E. coli*), for example, is the most common cause of BSIs in England, accounting for 18% of such infections [1]. Surveillance results from the 2007 Meropenem Yearly Susceptibility Test Information Collection (MYSTIC) show ongoing resistance across Europe for these Gram-negative pathogens [2]. *Acinetobacter *spp. also play an increasing role in healthcare-associated infections [1]. In Turkey, for example, susceptibility of *A.baumannii *to imipenem decreased from 80.4% in 2000 to 40.0% in 2006 and from 71.7% to 40.0% for meropenem during the same time period [3].

Doripenem is a carbapenem antibiotic with activity similar to imipenem and ertapenem against Gram-positive cocci, and similar to meropenem against Gram-negative pathogens [4]. Doripenem was approved in the European Union on July 25, 2008, for NP, including ventilator-associated pneumonia (VAP), cIAIs, and complicated urinary tract infections [5].

The COMParative Activity of Carbapenem Testing (COMPACT) Study was conducted to evaluate the in vitro activity of doripenem and comparator carbapenem antibiotics against recent Gram-negative clinical isolates; specifically *Pseudomonas *spp., *Enterobacteriaceae*, and other Gram negatives, including *A. baumannii*. This report focuses on the results from centres in Turkey and compares them with the general susceptibility pattern observed in COMPACT centres across Europe, the Middle East, and Africa.

## Methods

Isolates of *Pseudomonas aeruginosa*, *Enterobacteriaceae*, and other Gram negatives were collected prospectively between May 2008 and June 2009 from 80 centres across 16 countries in Europe, the Middle East, and Africa, including 10 centres in Turkey. Each centre was asked to prospectively collect 60 non-duplicate Gram-negative isolates. Isolates were obtained from intensive care unit (ICU) and non-ICU patients hospitalised with 1 of 3 types of infection: cIAI, BSI, or NP, including VAP. Collecting centres determined susceptibility of the isolates to doripenem, imipenem, and meropenem using E-test strips according to the manufacturer's guidelines.

The study protocol was reviewed and approved by an independent ethics committee. The study was conducted in accordance with the principles in the Declaration of Helsinki and was consistent with applicable regulatory requirements.

Isolates were batched by each centre and sent to a reference laboratory (Quotient Bioresearch Ltd., Fordham, UK) for species confirmation. The reference laboratory determined the minimum inhibitory concentration (MIC) of doripenem, imipenem, and meropenem for all isolates identified by each centre's E-test as imipenem- or meropenem-resistant, according to the 2009 Clinical and Laboratory Standards Institute (CLSI) breakpoints, or as doripenem non-susceptible by the US Food and Drug Administration (FDA) breakpoints (Table 1) [6]. The MIC was determined by both broth microdilution using CLSI methodology [7] and E-test according to the manufacturer's methodology. Limited susceptibility testing was performed for quality control purposes on each centre's E-test results by randomly selecting 10% of the susceptible isolates from each centre. FDA breakpoints were used for doripenem since CLSI breakpoints for doripenem were not available when the study was initiated. Breakpoints for *Enterobacteriaceae *were subsequently released in June 2010 [8]. CLSI breakpoints were used for imipenem and meropenem [9] since European Committee on Antimicrobial Susceptibility Testing (EUCAST) breakpoints were not available when the study was initiated. However, since EUCAST breakpoints for doripenem, imipenem, and meropenem are now available, they are also used for this data analysis [10].

**Table 1:** Breakpoints

Family/Genus (species)	FDA			CLSI						EUCAST								
	
	Doripenem			Imipenem/Meropenem			Doripenem/Imipenem/Meropenem*			Doripenem			Imipenem			Meropenem		
	
	S	I	R	S	I	R	S	I	R	S	I	R	S	I	R	S	I	R
*Pseudomonas aeruginosa*	≤ 2	-	-	≤ 4	8	≥ 16	≤ 4	8	≥ 16	≤ 1	2-4	≥ 8	≤ 4	8	≥ 16	≤ 2	4-8	≥ 16

*Enterobacteriaceae*	≤ 0.5	-	-	≤ 4	8	≥ 16	≤ 1	2	≥ 4	≤ 1	2-4	≥ 8	≤ 2	4-8	≥ 16	≤ 2	4-8	≥ 16

*Acinetobacter *spp.	≤ 1	-	-	≤ 4	8	≥ 16	≤ 4	8	≥ 16	≤ 1	2-4	≥ 8	≤ 2	4-8	≥ 16	≤ 2	4-8	≥ 16

*FDA *US Food and Drug Administration; *CLSI *Clinical and Laboratory Standards Institute; *EUCAST *European Committee on Antimicrobial Susceptibility Testing; *S *susceptible; *I *intermediate; *R *resistant

*As of June 2010

## Results

Ten centres in Turkey provided 596 eligible isolates. Patient demographics are shown in Table 2. By pathogen group, 297 (49.8%) were *Pseudomonas *spp., of which 98.7% were *P. aeruginosa *(49.2% of total); 240 (40.3%) isolates were *Enterobacteriaceae*, of which 47.9% were *E. coli *and 35.0% *Klebsiella pneumoniae *(19.3% and 14.1% of total, respectively); 59 (9.9%) were other Gram-negative bacteria, of which 89.8% were *A. baumannii *(8.9% of total) (Table 3).

**Table 2:** Isolates from Turkey by patient gender, age, location, and infection type

Number of Isolates	Gender		Age Group					Location		Infection Type		
**Species**	**F**	**M**	**0 to 2 yrs**	**3 to 5 yrs**	**6 to 17 yrs**	**18 to 64 yrs**	**> 64 yrs**	**ICU**	**Non-ICU**	**BSI**	**cIAI**	**NP**

*Acinetobacter baumannii*	21	32			2	36	15	41	12	21	6	26

*Acinetobacter haemolyticus*	1				1				1		1	

*Acinetobacter junii/johnsonii*	1	2				3		3		2		1

*Acinetobacter lwoffii*		1				1			1	1		

*Citrobacter freundii*	1					1			1		1	

*Enterobacter aerogenes*	3	6				7	2	6	3	1	2	6

*Enterobacter cloacae*	2	7	1			7	1	2	7	3	5	1

*Escherichia coli*	52	63	3	1	5	54	52	39	76	70	32	13

*Klebsiella oxytoca*	4	1	1		1	1	2	2	3	3		2

*Klebsiella pneumoniae*	33	51	5		1	42	36	47	37	38	9	37

*Kluyvera *sp.	1	1				1	1	2		1	1	

*Morganella morganii*	1	2	1			1	1		3	2	1	

*Pantoea *sp.		1				1			1		1	

*Proteus mirabilis*		2				1	1	1	1		1	1

*Pseudomonas aeruginosa*	113	180	18	3	7	168	97	160	133	90	40	163

*Pseudomonas putida*	2	1				1	2		3	1	2	

*Pseudomonas stutzeri*		1				1			1			1

*Raoultella terrigena*	1						1		1	1		

*Serratia marcescens*	2	6			1	3	4	6	2	7		1

*Stenotrophomonas maltophilia*		1				1			1			1

Total	238	358	29	4	18	330	215	309	287	241	102	253

*BSI *bloodstream infection; *cIAI *complicated intra-abdominal infection; *F *female; *ICU *intensive care unit; *M *male; *NP *nosocomial pneumonia

**Table 3:** Isolates from Turkey by specimen source

Number of Isolates	Source of Isolation				
**Species**	**Pulmonary Samples**	**Peritoneal Fluid**	**Blood**	**Others**	**Grand Total**

*Acinetobacter baumannii*	26		21	6	53

*Acinetobacter haemolyticus*				1	1

*Acinetobacter junii/johnsonii*	1		2		3

*Acinetobacter lwoffii*			1		1

*Citrobacter freundii*				1	1

*Enterobacter aerogenes*	6		1	2	9

*Enterobacter cloacae*	1	1	3	4	9

*Escherichia coli*	13	1	72	29	115

*Klebsiella oxytoca*	2		3		5

*Klebsiella pneumoniae*	37		39	8	84

*Kluyvera *sp.			1	1	2

*Morganella morganii*			2	1	3

*Pantoea *sp.				1	1

*Proteus mirabilis*	1			1	2

*Pseudomonas aeruginosa*	161	1	91	40	293

*Pseudomonas putida*			1	2	3

*Pseudomonas stutzeri*	1				1

*Raoultella terrigena*			1		1

*Serratia marcescens*	1		7		8

*Stenotrophomonas maltophilia*	1				1

Total	251	3	245	97	596

By type of infection, 42.4% of isolates were NP; 40.4%, BSI; and 17.1%, cIAI. Slightly more than half of the isolates came from patients in the ICU (51.8%), whilst 48.2% came from non-ICU patients (Table 2).

Of the 596 isolates, 187 (31.4%) were resistant to at least one carbapenem based on the E-test results reported by the collecting centre. Two hundred fifty-two isolates underwent reference laboratory confirmation of the centres' E-test results. Of the 91 determined by the centres to be susceptible to doripenem using current FDA breakpoints, 73 (80.2%) were confirmed as susceptible by the reference laboratory. Of the 161 determined to be non-susceptible to doripenem by the centres, 153 (95.0%) were confirmed as non-susceptible by the reference laboratory. For imipenem, of the 73 determined by the centres to be susceptible using CLSI breakpoints, 68 (93.2%) were confirmed as susceptible. Of the 179 determined by the centres to be non-susceptible (ie, intermediate or resistant) to imipenem, 173 (96.6%) were confirmed as non-susceptible by the reference laboratory. For meropenem, of the 98 determined by the centres to be susceptible using CLSI breakpoints, 80 (81.6%) were confirmed as susceptible. Of the 154 determined to be non-susceptible (ie, intermediate or resistant) to meropenem by the centres, 143 (92.9%) were confirmed as non-susceptible by the reference laboratory.

**Figure 1:**
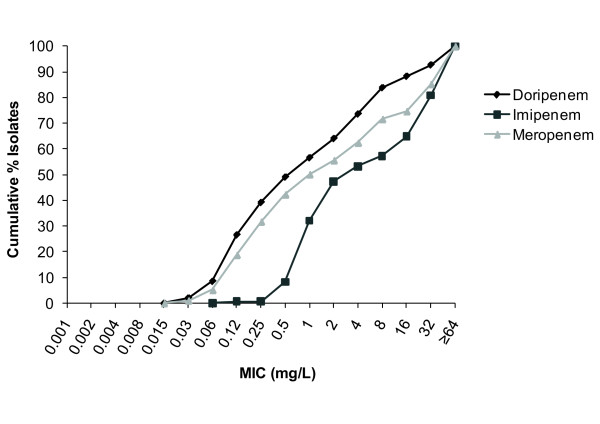
**Cumulative% minimum inhibitory concentration (MIC) distributions against *Pseudomonas aeruginosa *(N = 293)**.

For *P. aeruginosa*, the MIC_90 _was lowest for doripenem (32 mg/L) compared with ≥ 64 mg/L for both imipenem and meropenem (Table 4). Only 19.5% of *P. aeruginosa *isolates had a doripenem MIC > 4 mg/L compared with 25.8% and 30.9% for meropenem and imipenem, respectively (Figure 1, Tables 5, 6, 7). At MIC 2 mg/L (the FDA breakpoint for doripenem), 64.0% of *Pseudomonas *spp. were susceptible to doripenem, 48.2% to imipenem, and 56.2% to meropenem. At MIC ≤ 4 mg/L (the CLSI breakpoint for imipenem and meropenem), 74.1% were susceptible to doripenem, 53.9% to imipenem, and 63.0% to meropenem.

**Table 4:** Minimum inhibitory concentration (MIC) of all pathogens combined and pathogen groups from Turkey for doripenem, imipenem and meropenem

Turkey	N	MIC (mg/L)			
	
		Minimum	50%	90%	Maximum
**All pathogens**	596				
Doripenem		0.008	0.12	32	≥ 64
Imipenem		0.06	1	≥ 64	≥ 64
Meropenem		0.008	0.25	≥ 64	≥ 64

***Pseudomonas *spp**.	297				
Doripenem		0.03	1	32	≥ 64
Imipenem		0.12	4	≥ 64	≥ 64
Meropenem		0.03	1	≥ 64	≥ 64

***Enterobacteriaceae***	240				
Doripenem		0.008	0.03	0.12	32
Imipenem		0.12	0.25	0.5	≥ 64
Meropenem		0.008	0.03	0.12	≥ 64

**Other Gram negatives**	59				
Doripenem		0.03	8	≥ 64	≥ 64
Imipenem		0.06	32	≥ 64	≥ 64
Meropenem		0.06	32	≥ 64	≥ 64

**Table 5:** Isolates from Turkey: Doripenem MIC distribution

Number of isolates	Doripenem E-test MIC (mg/L)														
**Species**	**0.008**	**0.015**	**0.03**	**0.06**	**0.12**	**0.25**	**0.5**	**1**	**2**	**4**	**8**	**16**	**32**	**> 32**	**Grand Total**

*Acinetobacter baumannii*					1	1	1	5	3	7	7	3	7	18	53

*Acinetobacter haemolyticus*						1									1

*Acinetobacter junii/johnsonii*			1					1	1						3

*Acinetobacter lwoffii*														1	1

*Citrobacter freundii*		1													1

*Enterobacter aerogenes*		3	4		2										9

*Enterobacter cloacae*	1	3	4		1										9

*Escherichia coli*	9	59	37	4	1	3	2								115

*Klebsiella oxytoca*			3	2											5

*Klebsiella pneumoniae*	2	27	35	10	1	3	1		2		1	1	1		84

*Kluyvera *sp.		1						1							2

*Morganella morganii*				1	1	1									3

*Pantoea *sp.			1												1

*Proteus mirabilis*		1		1											2

*Pseudomonas aeruginosa*			6	19	53	37	29	22	22	28	30	13	13	21	293

*Pseudomonas putida*						1				2					3

*Pseudomonas stutzeri*					1										1

*Raoultella terrigena*			1												1

*Serratia marcescens*			4	1	3										8

*Stenotrophomonas maltophilia*										1					1

Grand Total	12	95	96	38	64	47	33	29	28	38	38	17	21	40	596

**Table 6:** Isolates from Turkey: Imipenem MIC distribution

Number of isolates	Imipenem E-test MIC (mg/L)											
**Species**	**0.06**	**0.12**	**0.25**	**0.5**	**1**	**2**	**4**	**8**	**16**	**32**	**> 32**	**Grand Total**

*Acinetobacter baumannii*			2		3	4	3	1	1	15	24	53

*Acinetobacter haemolyticus*			1									1

*Acinetobacter junii/johnsonii*	1						2					3

*Acinetobacter lwoffii*											1	1

*Citrobacter freundii*					1							1

*Enterobacter aerogenes*		1	5	3								9

*Enterobacter cloacae*		1	4	3	1							9

*Escherichia coli*		36	69	9	1							115

*Klebsiella oxytoca*		1	3	1								5

*Klebsiella pneumoniae*		26	44	7	2		1	1		2	1	84

*Kluyvera *sp.			1			1						2

*Morganella morganii*					1	2						3

*Pantoea *sp.				1								1

*Proteus mirabilis*		1			1							2

*Pseudomonas aeruginosa*		2		22	70	45	17	12	22	47	56	293

*Pseudomonas putida*			1		2							3

*Pseudomonas stutzeri*				1								1

*Raoultella terrigena*			1									1

*Serratia marcescens*			4	4								8

*Stenotrophomonas maltophilia*										1		1

Grand Total	1	68	135	51	82	52	23	14	23	65	82	596

**Table 7:** Isolates from Turkey: Meropenem MIC distribution

Number of isolates	Meropenem E-test MIC (mg/L)														
**Species**	**0.008**	**0.015**	**0.03**	**0.06**	**0.12**	**0.25**	**0.5**	**1**	**2**	**4**	**8**	**16**	**32**	**> 32**	**Grand Total**

*Acinetobacter baumannii*						1	1	4	3	5	5	5	8	21	53

*Acinetobacter haemolyticus*					1										1

*Acinetobacter junii/johnsonii*				1					1			1			3

*Acinetobacter lwoffii*														1	1

*Citrobacter freundii*		1													1

*Enterobacter aerogenes*		3	4	1	1										9

*Enterobacter cloacae*	1	1	6		1										9

*Escherichia coli*	3	50	39	16	3	2	2								115

*Klebsiella oxytoca*		1	2	2											5

*Klebsiella pneumoniae*	1	20	32	18	4	2	2		1	1			2	1	84

*Kluyvera *sp.		1						1							2

*Morganella morganii*			1	1	1										3

*Pantoea *sp.		1													1

*Proteus mirabilis*			1		1										2

*Pseudomonas aeruginosa*			3	12	40	38	31	23	16	20	27	9	31	43	293

*Pseudomonas putida*								2	1						3

*Pseudomonas stutzeri*						1									1

*Raoultella terrigena*			1												1

*Serratia marcescens*		1	2	3	1	1									8

*Stenotrophomonas maltophilia*									1						1

Grand Total	5	79	91	54	53	45	36	30	23	26	32	15	41	66	596

**Figure 2:**
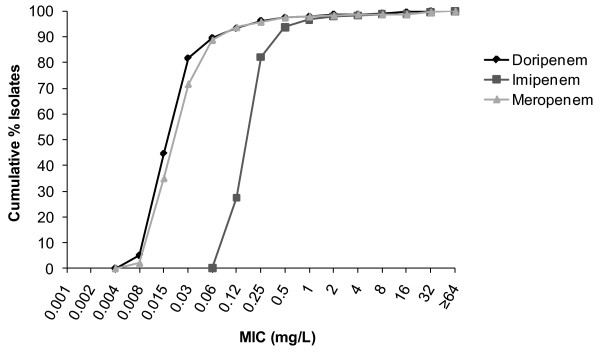
**Cumulative% minimum inhibitory concentration (MIC) distributions against *Enterobacteriaceae *(N = 240)**.

**Figure 3:**
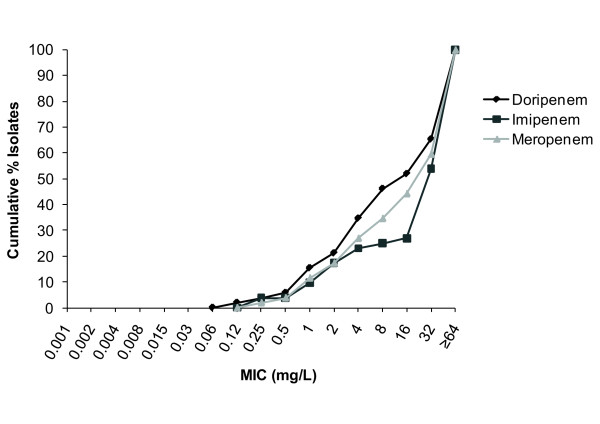
**Cumulative% minimum inhibitory concentration (MIC) distributions against *Acinetobacter baumannii *(N = 53)**.

For *Enterobacteriaceae*, doripenem and meropenem were equally active (MIC_90 _0.12 mg/L) and at least four-fold more active than imipenem (MIC_90 _0.5 mg/L; Figure 2). At MIC 0.5 mg/L (the FDA breakpoint for doripenem against *Enterobacteriaceae*), 97.5% were susceptible to doripenem, 93.75% to imipenem, and 97.5% to meropenem. At MIC ≤ 4 mg/L (the 2009 CLSI breakpoint for imipenem and meropenem against *Enterobacteriaceae*), 98.75% were susceptible to doripenem, 98.33% to imipenem, and 98.75% to meropenem. At MIC ≤ 1 mg/L (the new breakpoint for imipenem and meropenem, as well as doripenem, against *Enterobacteriaceae *established by CLSI in June 2010), 97.92% were susceptible to doripenem, 96.67% to imipenem and 97.92% to meropenem. Also at MIC ≤ 1 mg/L, 100% of *E. coli *and 94.1% of *K. pneumoniae *were susceptible to each of the three carbapenems. The MIC_90 _for all three carbapenems against other Gram-negative isolates, including *A. baumannii *(Figure 3), was ≥ 64 mg/L.

Based on the newly established EUCAST breakpoints for carbapenems (Table 1), 43.4% of *Pseudomonas *spp. and 2.1% of *Enterobacteriaceae *isolates were deemed doripenem non-susceptible according to the E-test MIC results from the collecting centres (Table 8). Regarding *Pseudomonas *spp., 46.1% were non-susceptible to imipenem and 43.8% to meropenem, whilst 2.1% and 1.7% of *Enterobacteriaceae *were non-susceptible to imipenem and meropenem, respectively.

**Table 8:** Susceptibility* of all pathogens from Turkey: Combined pathogens and pathogen groups for doripenem, imipenem, and meropenem

Turkey	N	Percentage of isolates		
	
		Susceptible	Intermediate	Resistant
**All pathogens**	596			
Doripenem		69.5	11.1	19.5
Imipenem		68.1	3.4	28.5
Meropenem		69.8	9.7	20.5

***Pseudomonas *spp**.	297			
Doripenem		56.6	17.5	25.9
Imipenem		53.9	4.0	42.1
Meropenem		56.2	15.8	28.0

***Enterobacteriaceae***	240			
Doripenem		97.9	0.8	1.3
Imipenem		97.9	0.8	1.3
Meropenem		98.3	0.4	1.3

**Other Gram negatives**	59			
Doripenem		18.6	20.3	61.0
Imipenem		18.6	10.2	71.2
Meropenem		22.0	17.0	61.0

*Based on current European Committee on Antimicrobial Susceptibility Testing breakpoints

## Discussion

The COMPACT surveillance study involving 10 centres in Turkey demonstrated that doripenem has similar or slightly better activity than imipenem and meropenem against *Pseudomonas *spp., *Enterobacteriaceae*, and other Gram-negative pathogens.

Compared with the other European, Middle Eastern, and African countries that participated in COMPACT, Turkey had a high rate (31.4%) of combined imipenem resistance, meropenem resistance, or doripenem non-susceptibility [11]. This rate in Turkey was second only to Russia (33.6%), and followed by Egypt (28.3%), Spain (23.6%), Italy (23.2%) and the remainder of the 16 countries involved.

In Turkey, doripenem was the most active of the 3 carbapenems against *Pseudomonas *spp. The non-susceptible (intermediate and resistant) rate of 46.1% for *Pseudomonas *spp. (98.7% *P. aeruginosa*) to imipenem observed in this study is higher than the 32% observed from 2004 to 2006 with VAP isolates [12]. The non-susceptible rate for imipenem in this study also is higher than the 16.1% observed from 2000 to 2002 for *P. aeruginosa *in both coronary and surgical ICU patients [13].

Against *Enterobacteriaceae*, doripenem and meropenem were equally active and at least four-fold more active than imipenem. These results are consistent with susceptibility data from the United Kingdom and Ireland for 2001 to 2006 [14]. In addition, the activity of imipenem and meropenem observed in Turkey in COMPACT was very similar to the susceptibility rate of 97.6% observed by Korten et al. for imipenem against all *Enterobacteriaceae *isolates from Turkey between 2000 and 2003 [15].

None of the 3 carbapenems showed good activity against *A. baumannii*. This is not surprising given the high rates of resistance observed over the past decade in Turkey [12,13]. The MIC_90 _for *A. baumannii *was several-fold higher in this study than the MIC_90 _> 8 and > 16 mg/L observed for imipenem and meropenem, respectively, in the SENTRY Antimicrobial Surveillance Program from 2000 to 2006 in Ankara and Istanbul, Turkey [3].

The COMPACT surveillance study also was carried out in 6 Asia-Pacific countries. As in the 10 centres in Turkey and the 80 centres throughout Europe, the Middle East, and Africa, doripenem was the most active of the carbapenems tested against Asia-Pacific isolates [16]. The MIC_90 _against all Asia-Pacific isolates was 8 mg/L for doripenem compared with 32 mg/L for Turkey. The mean MIC_90 _against all isolates for imipenem and meropenem (both ≥ 64 mg/L) was the same for Turkey and the Asian-Pacific countries.

## Conclusions

In conclusion, the carbapenems possess good activity against the Gram-negative isolates included in this study, including *Pseudomonas *spp. and *Enterobacteriaceae*, among the 10 collecting centres in Turkey. Although the rate of combined imipenem resistance, meropenem resistance, or doripenem non-susceptibility was high in Turkey and second only to Russia, doripenem was the most active carbapenem against *P. aeruginosa*, was equally active to meropenem, and was more active than imipenem against *Enterobacteriaceae*.

## Competing interests

This work was supported by Janssen EMEA. The decision to submit this article for publication was made by Janssen and the authors. No financial support or honorarium was given to the non- Janssen authors for the development of this manuscript. H. Baris and J. Laeuffer are employees of Janssen EMEA. These authors were not awarded any additional support outside of their salaries for their participation in this study. Editorial assistance was provided by Phase Five Communications Inc., which was funded by Janssen EMEA.

## Authors' contributions

HL was involved in the trial design as well as data collection and analysis. He also participated in drafting the manuscript, and reviewed and approved the final draft. NC was involved in the data collection and analysis. He also participated in drafting the manuscript, and reviewed and approved the final draft. MC was involved in the data collection and analysis. He also participated in drafting the manuscript, and reviewed and approved the final draft. HK was involved in the data collection and analysis. He also participated in drafting the manuscript and reviewed the final draft. HB was involved in the trial design and data analysis. He also participated in drafting the manuscript, and reviewed and approved the final draft. JL was involved in the trial design and data analysis. He also participated in drafting the manuscript, and reviewed and approved the final draft. All authors read and approved the final manuscript.

### COMPACT Turkey study investigators

F. Akata and Z. Yuluğkural (Trakya University, Edirne, Turkey); C. Ayaz (Dicle University, Diyarbakir, Turkey); G. Çelebri and F. Cömert (Karaelmas University, Zonguldak, Turkey); D. Gerçeker (Ankara University, Ankara, Turkey); Z. Gülay (Dokuz Eylül University, Izmir, Turkey); M. Günaydin (Ondokuzmayis University, Samsun, Turkey); A. Kaya and G. Ersöz (Mersin University, Mersin, Turkey); S. Özer and N. Benzononana (Dr. Lütfü Kardar Hospital, Istanbul, Turkey); and Y. Taşova and A. Yaman (Çukorova University, Adana, Turkey).

## Pre-publication history

The pre-publication history for this paper can be accessed here:

http://www.biomedcentral.com/1471-2334/12/42/prepub
